# Supplementary material for: Study of Effector CD8+ T Cell Interactions with Cortical Neurons in Response to Inflammation in Mouse Brain Slices and Neuronal Cultures
Source: Int J Mol Sci. 2023 Feb 6;24(4):3166. doi: 10.3390/ijms24043166 (PMC9960285; doi:10.3390/ijms24043166)
Supplement: Supplementary file 1 [file ijms-24-03166-s001.zip › ijms-2163851-supplementary.pdf]

Supplementary Materials

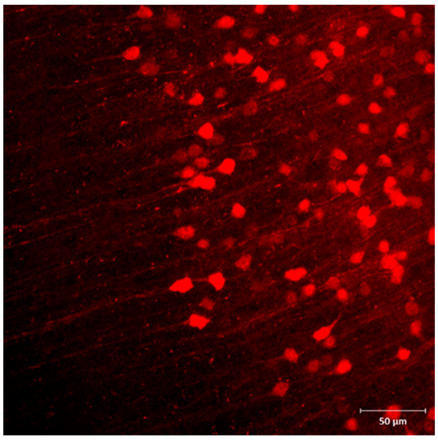

Supplementary Figure S1. Intact cortical neurons in an acute brain slice after sectioning. Confocal image of an acutely isolated brain slice from a 2 week old Thy1.2-HcRed pup. Cortical neurons expressed cytosolic red fluorescence protein HcRed1. This data is related to Figure 3.

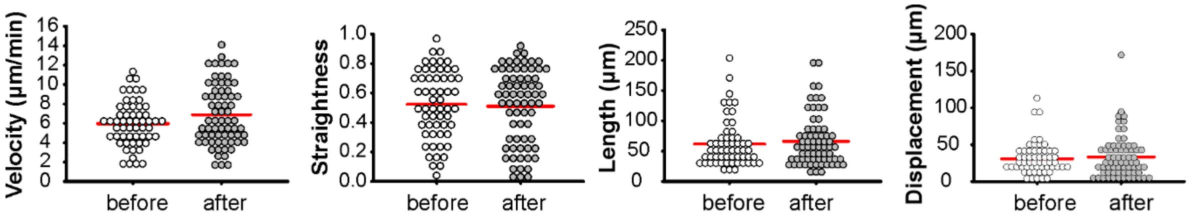

Supplementary Figure S2. T cells do not change migrating pattern without cytokine application. WT T cells were co-cultured with neuronal cells and being recorded by confocal microscopy in real time. The videos were acquired for first 45 min (before) and further recorded for another 45 min (after) without cytokine application as a control experiment related to Figure 3.

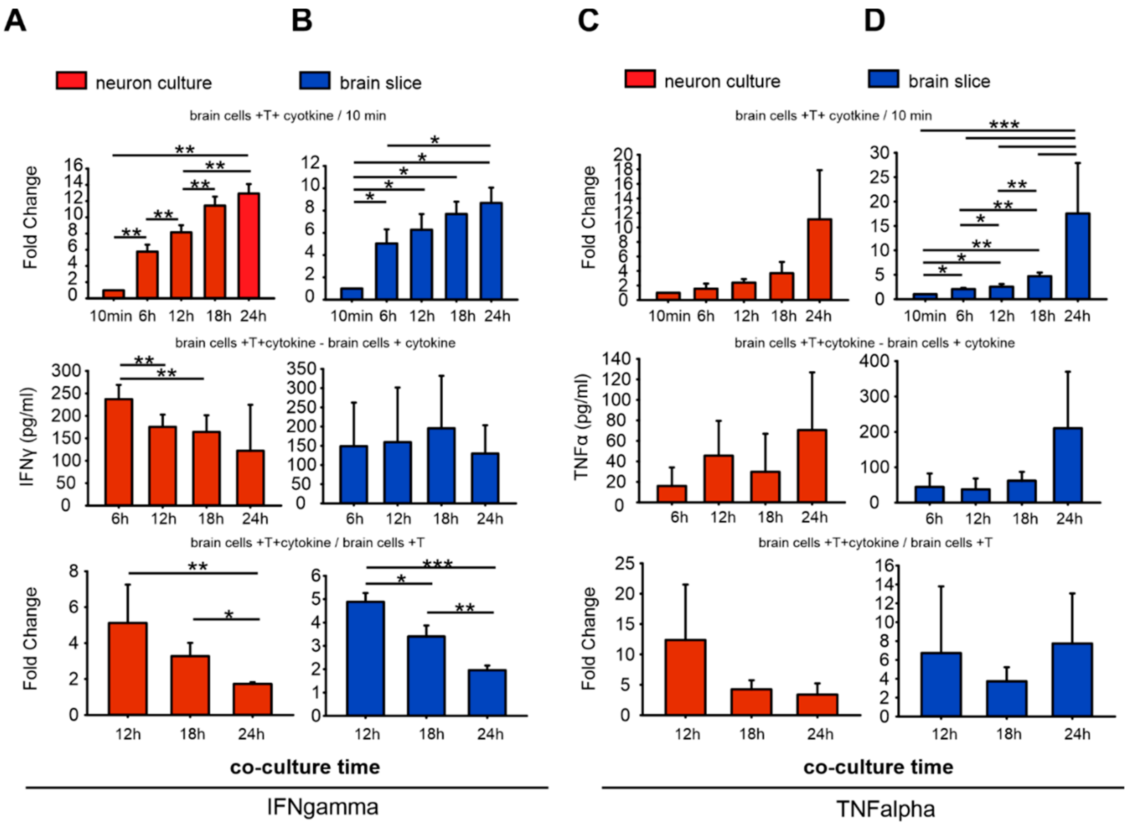

Supplementary Figure S3. Pro-inflammatory cytokine release in T cell-neuronal cells and T cells-brain slices co-culture. T cells were co-cultured with cortical cultures *in vitro* and *ex vivo*. The supernatants were collected at different time points for ELISA cytokine measurements. This data is related to Figure 4. * $p < 0.05$; ** $p < 0.01$; *** $p < 0.001$

Condition		R^2 linear regression	R^2 exponential growth
IFNg	Neurons only	nd	nd
	Neurons + T cells	0.91	0.79
	Neurons + cytokine	0.99	-0,21
	Neurons + T cells + cytokine	0.95	-1.07
TNFa	Neurons only	nd	nd
	Neurons + T cells	0.76	0.99
	Neurons + cytokine	0.98	0.99
	Neurons + T cells + cytokine	0.97	0.97
IFNg	Brain slices only	nd	nd
	Brain slices + T cells	0.91	0.99
	Brain slices + cytokine	0.94	nd
	Brain slices + T cells + cytokine	0.94	nd
TNFa	Brain slices only	nd	nd
	Brain slices + T cells	0.76	0.99
	Brain slice + cytokine	0.74	0.99
	Brain slices + T cells + cytokine	0.70	0.99

Supplementary Table S1. R^2 values of curve fittings of IFNγ and TNFα release. Data for all conditions were fitted with both linear regression and an exponential growth function. R^2 values are shown as a measure of quality. The closer R^2 value to 1 is, the better the fit is. It can be seen that a linear regression is better suited to describe the release of IFNg while the release of TNFa is better described with an exponential grows function. This data is related to Figure 3F.

Supplementary Video S1. T cells migration on neuronal layer. Effector CD8+ T cells (green) isolated from CD8cre-τGFP mice were co-cultured with cortical neurons *in vitro*. Neurons were lentivirally infected with mitochondria marker 4mts-mscarlet I (red). T cell migration were recorded by live cell confocal imaging. This data is related to Figure 3A.

Supplementary Video 2. T cells migration on brain slice. Effector MOG-specific T cells isolated from CD8cre-τGFP mice were co-cultured with *ex vivo* brain slices isolated from a 2 week old Thy1.2-HcRed pup. Cortical neurons expressed cytosolic red fluorescence protein HcRed1. T cell migration were recorded by live cell confocal imaging. This data is related to Figure 3F.
